# Atraumatic restorative treatment in São Paulo, SP, schools: implementation strategies and preliminary impact

**DOI:** 10.1590/1807-3107bor-2026.vol40.091

**Published:** 2026-11-09

**Authors:** Fábio Carneiro MARTINS, Daniela Prócida RAGGIO, Laura Regina Antunes PONTES, Mariana GABRIEL, Antônio Carlos FRIAS, Doralice Severo CRUZ, Ana Estela HADDAD, Fernanda Campos de Almeida CARRER

**Affiliations:** (a) Universidade de Brasília - UnB, School of Health Sciences, Department of Dentistry, Brasília, DF, Brazil.; (b) Universidade de São Paulo - USP, School of Dentistry, Department of Orthodontics and Pediatric Dentistry, São Paulo, SP, Brazil.; (c) Faculdade Galileu, School of Dentistry, Botucatu, SP, Brazil.; (d) Universidade de São Paulo - USP, School of Dentistry, Department of Social Dentristry, São Paulo, SP, Brazil.; (e) Coordenação Nacional de Saúde Bucal, Brasília, DF, Brazil.

**Keywords:** Dental Atraumatic Restorative Treatment, Dental Caries, Oral Health, Health Policy

## Abstract

This study aimed to describe and analyze the implementation of the “ARTSUS-SP” strategy in the city of São Paulo, which incorporated Atraumatic Restorative Treatment (ART) into the School Health Program to expand and improve students’ access to dental care through treatments provided in public primary schools.

**Methods:**

Data analysis was guided by the Sustainable Oral Health Framework. In the qualitative phase, the research teams examined meeting minutes and speeches from the Specialist Committee, comprising professors (n = 6), graduate students (n = 10), managers (n = 3), and supporting health and education professionals (n = 40). This analysis focused on training stages, strategy adoption, and facilitators and barriers to implementation. In the quantitative phase, a descriptive analysis was conducted considering the number of participating Basic Health Care Units, children treated, teeth restored, and reported pulp exposure.

**Results:**

The pilot project and training program were well accepted and aligned with team needs. Key aspects included the hybrid training course and the incorporation of local contextual factors. Quantitative data demonstrated an increase in coverage, with 10,300 children receiving care and 23,638 decayed teeth restored over a six-month period.

**Conclusion:**

Implementation was supported by motivated leadership and well-trained teams, resulting in a positive impact on access to dental care. However, barriers such as high staff turnover and resource limitations remain critical challenges to long-term sustainability.

## Introduction

The World Health Organization estimates that dental caries is among the main public health problems and represents the most widespread non-communicable disease worldwide. The age-standardized prevalence of untreated dental caries in permanent teeth was 27,500 cases per 100,000 population, affecting approximately 3.69 billion people in 2021.^1^ More than one-third of the global population lives with untreated dental caries.^2^ In Brazil, 53.17% of children aged 5 years are affected by caries, and at age 12 years, 49.88% present the same condition.^3^

Access to health services refers to the timely use of health care that meets individual needs for achieving the best possible health outcomes and can be understood as a factor that ensures quality of life, dignity, and health for populations.^4,5^ In oral health, access corresponds to the availability and effective utilization of preventive and restorative care, which directly influences individuals’ ability to eat, speak, and socialize without pain or discomfort. Since 2004, Brazil has implemented a National Oral Health Policy that expanded dental service coverage, thereby improving access; however, many challenges persist.^6-8^

One alternative to increase and improve access is the implementation of Atraumatic Restorative Treatment (ART) as a health strategy or policy. ART is a minimally invasive technique, aligned with the modern philosophy of health care and consisting of the selective removal of carious dentin using hand instruments, followed by the restoration of cavitated, active, and retentive lesions with high-viscosity glass ionomer cement. As a clinical strategy, ART stands out for its safety, high success rates, and strong patient acceptability. As a public health policy strategy, it is relevant for expanding access to restorative care, since it can be performed in alternative settings.^9^

Despite strong clinical evidence supporting ART, few studies focus on its use as a policy implementation strategy.⁸ Although ART is supported by the scientific literature and recommended by the World Health Organization (WHO), resistance among professionals and managers remains common, primarily due to misconceptions about lower effectiveness, lack of training, false perceptions of increased workload, limitations in supplies, and institutional or cultural barriers; consequently, ART remains underutilized in health services.^9^

In the city of São Paulo, Oral Health Teams annually conduct health promotion and prevention activities in public schools through the School Health Program. During these activities, risk classification is performed using a demand organization index developed by the state of São Paulo.^10^ In this system, group “A” comprises individuals with all sound teeth; group “B” includes those with at least one restored tooth; “C” includes those with at least one chronic lesion or provisional restoration; group “D” includes those with at least one active white spot lesion; group “E” includes those with at least one single-surface, active cavitated carious lesion without pulpal involvement; group “F” includes those with at least one multiple-surface active cavitated carious lesion without pulpal involvement; and “G” includes those with at least one tooth presenting pulpal exposure, fistula, abscess, or spontaneous pain.

The Oral Health Teams used this index in 2017 to report to the municipal health department that more than 80,000 children in the city had at least one active cavitated carious lesion requiring intervention. At that time, after application of the index, children received referral letters to take home, guaranteeing priority treatment in more than 400 Basic Health Care Units (UBS) throughout the city. However, most parents or guardians did not bring their children for treatment, resulting in high absenteeism. Preliminary studies conducted in other Brazilian states have warned that absenteeism rates in similar contexts may exceed 90%.^11,12^ No published data are available for this period in the city of São Paulo.

Given this scenario, which resulted in disease progression and negative impacts on school performance, pain, and suffering,^13,14^ the implementation of a program called “ARTSUS-SP” was proposed. The project was designed to be implemented within the School Health Program so that the Oral Health Teams could provide care for these children directly at schools using ART,^15,16^ supported by robust and up-to-date scientific evidence.^17-20^

ARTSUS-SP was planned and implemented with technical assistance from a specialist committee composed of faculty members and graduate students from the School of Dentistry of the University of São Paulo and the Evidence and Economic Analysis Center of the same institution, and mobilization of municipal health management, in 2018 and early 2019. Implementation occurred through a pilot project, semi-presential training (theoretical and preclinical), and impact evaluation, the stages of which are described and analyzed in this study. Data collection and analysis were related to these stages and were conducted during the same period. Therefore, this study aimed to describe and analyze factors related to the implementation process of ART as a strategy to address dental caries in the Oral Health Care Network in São Paulo, Brazil (ARTSUS-SP strategy).

## Methods

### Study design

This study was designed as a case study^10^ based on an exploratory mixed-methods approach combining quantitative and qualitative methods, as illustrated in Figure 1. It describes and analyzes the different implementation stages of a municipal strategy called ARTSUS-SP within the School Health Program in the city of São Paulo, São Paulo State, located in southeastern Brazil.

**Figure 1:**
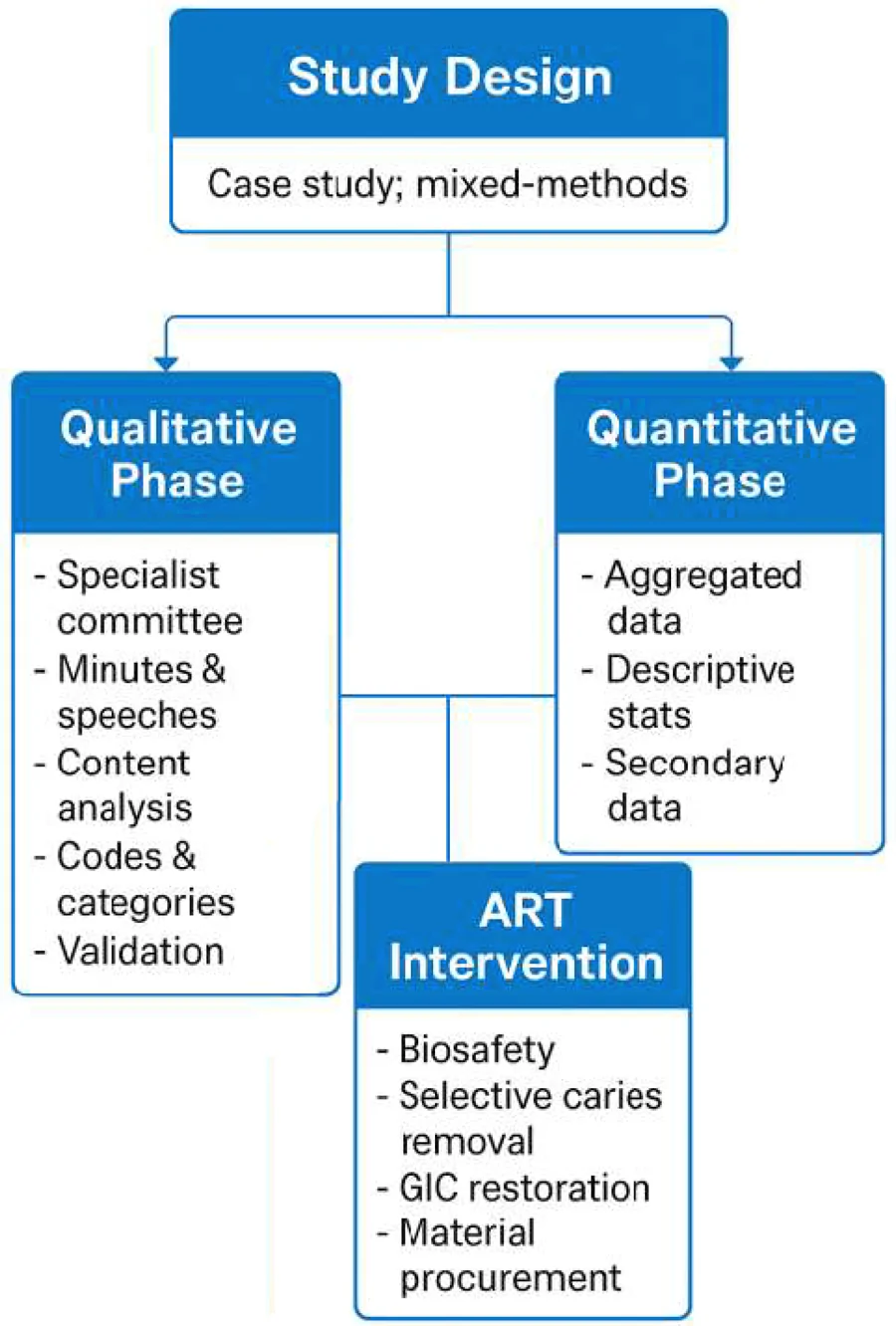
Schematic diagram illustrating the study design.

### Framework

A framework was adopted to guide the implementation of sustainable oral health interventions.^21^ The Sustainable Oral Health Framework is grounded in principles of health promotion, equity, and sustainability, integrating social, environmental, and organizational factors that influence oral health outcomes. It draws on established theories, including the PRECEDE–PROCEED model, the Diffusion of Innovation, and behavior change theories, to guide the implementation and sustainability of oral health interventions.^21^

The framework describes how different implementation stages can affect the sustainability of strategies, programs, and policies, emphasizing three domains: a) training dissemination; b) strategy adoption; and c) implementation, as described in this study. Training dissemination involves the planning and delivery of educational activities to ensure that professionals acquire the necessary knowledge and skills. Strategy adoption refers to the decision-making processes and organizational commitment required for professionals and institutions to integrate the new intervention into routine practice. Implementation encompasses the practical execution of the strategy within services, including monitoring adherence, addressing barriers, and supporting fidelity to the intended procedures.

In addition, the framework describes a fourth stage—practice improvement—involving service evaluation and economic analysis, which was not the focus of the present study. The framework guided both data collection and analysis. The first three domains informed the qualitative phase, while the implementation stage also supported the quantitative phase.

### Participants

The Specialist Committee comprised professors (n = 6), graduate students (n = 10), managers (n = 3), and designated health and education support professionals (n = 40) involved in implementation. Children treated, oral health professionals, and schools involved were not considered participants, since the procedures were conducted within the context of a public policy decision aligned with the guidelines of the Brazilian Unified Health System (SUS), the School Health Program (Programa Saúde na Escola), and routine service protocols. Nonetheless, it should be pointed out that schools that had previously agreed to implement oral health actions through the School Health Program in collaboration with the São Paulo Municipal Health Department were those that received the activities during the implementation. The number of participating schools could not be determined, since the data on procedures performed were recorded only by the regional health centers.

### Design, data collection, and analysis

Data were collected from multiple sources using a mixed-methods approach based on an exploratory quantitative–qualitative model,^22^ as described in greater detail in Table 1 and in the following sections entitled Qualitative Phase and Quantitative Phase.

**Table 1:** Characteristics of the categories and codes described and analyzed according to the stages defined by the framework for the implementation of sustainable oral health interventions, with a focus on Artsus-SP.

Stage	Categories	Considered aspects (Codes)	Sources of information
Training dissemination	Pilot project and preparatory courses	Pilot project: champions, flexibility, satisfaction	Discourse analysis of the Specialist Committee based on meeting minutes from specialists involved in the process.
		Preparatory courses: compatibility with team needs, accessibility	
Strategy adoption	Planning	Evidence-based decision-making	Discourse analysis of the Specialist Committee based on meeting minutes from specialists involved in the process.
		Leadership, usefulness, adaptability	
Implementation	First year of ARTSUS-SP	Facilitators: effectiveness, feasibility	Quantitative data provided by the Oral Health Coordination (after the first months of policy implementation);
		Barriers: managerial and oral health team turnover, resistance, low fidelity	discourse analysis of the Specialist Committee based on meeting minutes from specialists involved in the process.

### Qualitative phase

This phase was conducted using content analysis,^23^ in which the speeches of Specialist Committee members and the meeting minutes were analyzed. A total of six meetings, each lasting approximately 1 hour and 30 minutes, were examined. No questions were developed, nor was a focus group conducted; the analysis was based exclusively on the meeting minutes and the speeches delivered by the Specialist Committee during strategy planning meetings.

Categorization was performed independently by two researchers. After floating reading, followed by word-by-word and sentence-by-sentence analysis, codes, categories, and subcategories were identified. These were then compared and discussed until a consensus was reached. Following consensus, the codes were classified into the different domains of the framework, as described in Table 1. Both the meeting minutes and the codes were validated by the Specialist Committee participants to enable triangulation of the analyses.

### How ART was performed

As outlined in the Introduction, approximately 80,000 children in São Paulo had at least one tooth with an active, cavitated, and retentive carious lesion. According to oral health guidelines,^10^ all children, regardless of risk group, received oral hygiene and dietary instructions. Children classified in groups B, C, and D received topical fluoride application and ART sealants. Children classified in groups E and F, with at least one cavitated carious lesion, without pulpal involvement in primary and permanent teeth, could be treated with ART restorations at school, within a program planned and implemented in partnership with this research group. Children requiring extractions or endodontic treatment were referred to local health centers. These protocols are detailed in Table 2.

**Table 2:** Protocols carried out.

Group	Situation identified	Treatment
A	No history of carious lesions: only sound teeth	Oral hygiene and dietary guidance
B	Presence of at least one restored tooth	Oral hygiene and dietary guidance
C	Presence of inactive carious lesion and/or provisional restoration	Hygiene and dietary guidance
D	Presence of carious lesion in enamel (active white spot)	Hygiene and dietary guidance; topical application of fluoride gel; application of ART sealants in erupting molars with active noncavitated lesions
E	Presence of occlusal cavitated carious lesions without pulp involvement	Oral hygiene and dietary guidance; topical application of fluoride gel; ART
F	Presence of carious lesion on proximal surface, incisal angles, or cervical surfaces without pulp involvement	Oral hygiene and dietary guidance, topical application of fluoride gel; ART
G	Suspected pulpal or periapical involvement (pulpitis, fistula, exposed pulp, abscess, residual focus or pain)	Oral hygiene and dietary guidance; topical application of fluoride gel; application of ART sealants; ART; referral to the health unit for tooth extraction, endodontic treatment, or other complex procedures

ART procedures were independently performed by SUS professionals in schools. This project evaluated only the impact of implementation and supported professional training, during which standardized biosafety protocols and restorative technique steps were established:

#### Biosafety

Schools were notified in advance and provided a well-lit room with a sturdy table covered with a mattress, a nearby sink, and an auxiliary desk for instruments. Instrument sterilization was performed at the Primary Health Care Unit (PHCU) in sealed metal boxes, in accordance with sanitary regulations. Waste was collected in white hospital-type bags and discarded at the PHCU. Following each procedure, surfaces were disinfected with 70% alcohol and covered with disposable paper. Handwashing was performed before receiving each patient, and disposable gloves, masks, and caps were changed after each procedure.

#### ART technique

- Cavity preparation: isolation with cotton rolls, cleaning with moist and dry cotton; selective caries removal using excavators—complete removal of infected dentin from the cavity walls while preserving firm dentin on the cavity floor in shallow or moderate lesions and softened dentin in deep lesions; placement of a matrix and wedge when indicated; conditioning with GIC liquid for 10 s, rinsing and drying.
- Glass ionomer cement (GIC) preparation and application: performed according to the manufacturer’s instructions (powder scoop:liquid drop 1:1), manipulated until a firm consistency was achieved, and inserted within 90 s.
- Restoration: incremental insertion; finger pressure with petroleum jelly; removal of the matrix and wedge after 5 min; occlusal adjustment; removal of excess material; surface protection with petroleum jelly; and instruction to avoid food intake for 1 hour.

Initially, Maxion was used as the restorative material. During implementation, management modified procurement specifications, as recommended by the research group of this study, to incorporate high-viscosity glass ionomer cements. Following an analysis and selection process conducted by the Municipal Health Department, Ketac Molar glass ionomer cement was adopted.

## Quantitative phase

Descriptive statistics were applied to quantitative data to monitor and assess the impact of implementation. The variables analyzed included the number of participating health care units; their geographic distribution across city regions, based on the official Health Technical Supervision divisions (Central, East, North, West, Southeast, and South); the number of students involved; and the number of teeth treated, including reports of pulp exposure during procedures as documented by the operators. These data were provided by the Oral Health Coordination of the Municipal Health Department after the first six months of implementation and consisted of secondary data aggregated by health regions. It is important to note that the research team did not have access to the primary databases, since only consolidated regional data were made available, which limited the possibility of conducting analytical statistical analyses.

## Ethical considerations

This research was approved by the Research Ethics Committees of the São Paulo Municipal Health Department (protocol no. 3,144,396) and the School of Dentistry of the University of São Paulo (protocol no. 3,141,308). In the quantitative phase, secondary data provided by the Municipal Oral Health Coordination of the São Paulo Municipal Health Department were used. It should be emphasized that the treatments were implemented as a municipal strategy (public policy), independent of the research, and that this study described and analyzed data related to the development and impact of this strategy; therefore, the procedures were not conducted as part of this study. However, procedures were performed only after a specific informed consent form for the School Health Program was signed, in accordance with pre-established guidelines. This process constitutes an inherent component of the routine of oral health services in the city of São Paulo.

In the qualitative phase, meeting minutes from a specialist committee, composed of professionals from the School of Dentistry of the University of São Paulo and the São Paulo Health Department, as described in the Context and Participants sections, were analyzed. For this phase, participants provided their free and informed consent for the use of the minutes and their analysis by the research team.

## Results

The implementation of ARTSUS-SP followed the stages outlined in Figure 2, which illustrates a timeline of the activities carried out. The results were described and analyzed based on Table 1, which presents the domains of the framework applied: a) training dissemination; b) strategy adoption; and c) implementation.

**Figure 2:**
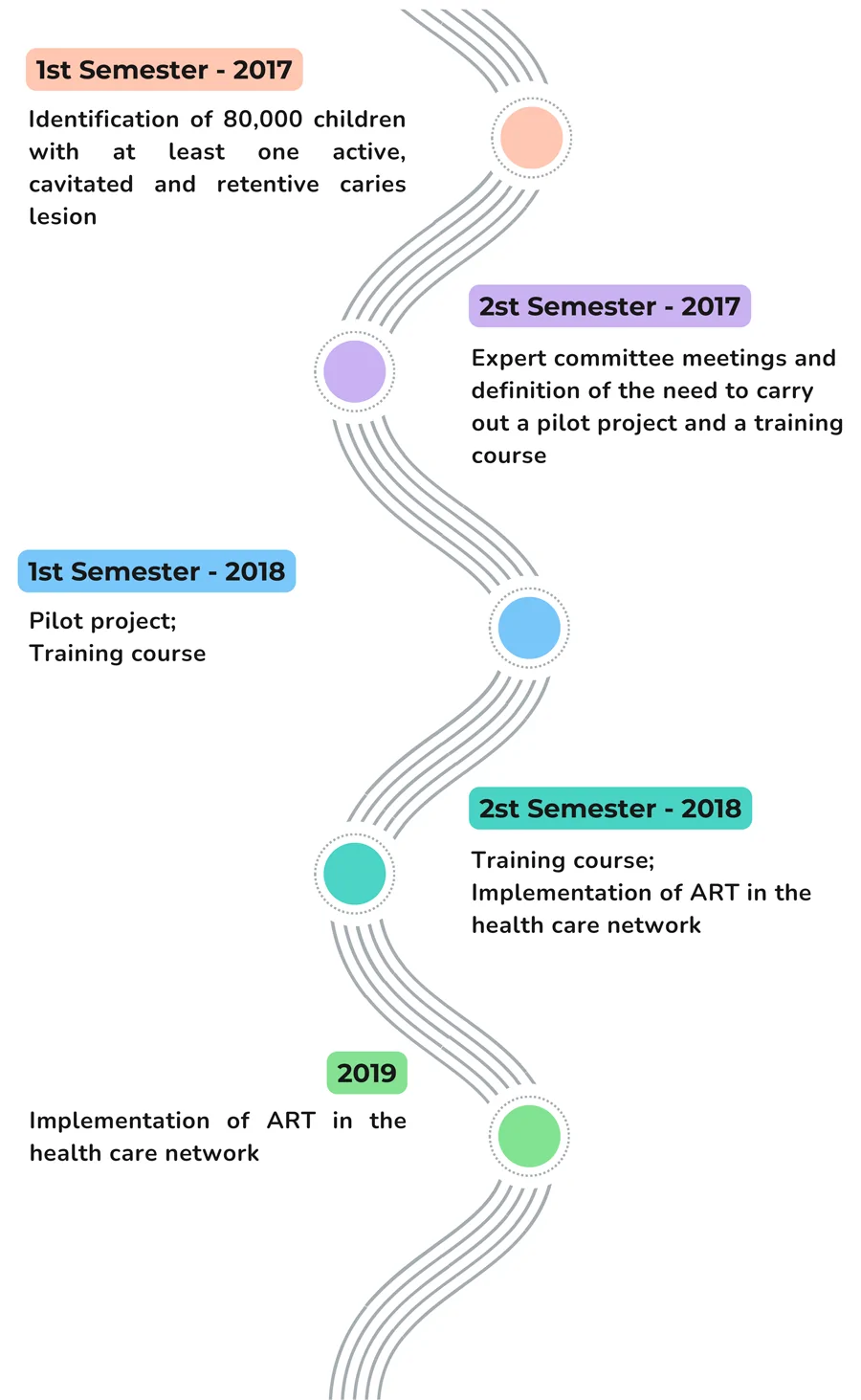
Timeline.

Regarding training dissemination, the Specialist Committee identified the need to conduct a pilot project before extending the training to all teams involved in strategy implementation. The pilot project was conducted at EMEI Gilberto Nascimento School, located in the area covered by the Boa Vista Basic Health Care Unit, where the School of Dentistry of the University of São Paulo conducts community-based internships within SUS. Following the pilot project, lessons learned were identified, and the work process was revised to operationalize a model that ensured user safety, worker ergonomics, and biosafety, as shown in Table 3.

**Table 3:** Pilot Project: identified results and participant quotes.

Considered aspects (codes)	Identified results	Participant quotes
Flexibility	Need for: (1) prior situational diagnosis to identify children with caries; (2) organized school records on student enrollment; (3) naturally lit spaces; (4) separate rooms for clinical care; (5) collaboration and effective communication with school management, teachers, and regional education board; (6) availability of mats in schools; and (7) low tables allowing ergonomic posture for professionals.	“As long as it’s scheduled, it doesn’t interfere with pedagogical activities.” (Participant 1)
		“We contact parents through the school office to explain the health activities, and they trust us a lot, especially because we have been doing this work for over 30 years.” (Participant 2)
		“The workflow organization was excellent. Since risk classification was performed in advance, health and school professionals knew which children needed care. Among those eligible for school-based treatment, we prioritized the most severe cases, followed by the simpler ones. Only children with deep caries were referred to the health unit. Teachers supported communication with the parents.” (Participant 3)
		“I believe schools should receive this type of intervention, since parents are receptive and often have great difficulty taking their children to the health center.” (Participant 4)
		“One factor that really helped was having a classroom consistently available for ART, with low tables, good natural lighting, mats, and a location away from busy hallways.” (Participant 3)
Satisfaction	The pilot project overcame initial resistance among the Oral Health Teams, education professionals, and children.	“I pick up the child from the classroom and start explaining what the dentist will do. Then I wait for the procedure and take the child back to class... they already know the space, and that reassures them.” (Participant 2)
Champions	Key actors who promoted and sustained implementation: school teachers and principals;	“I believe schools should receive this kind of work, because parents are receptive and have great difficulty taking their children to the health center.” (Participant 1)
	Oral Health Teams; professors and students from the School of Dentistry of the University of São Paulo.	“We were able to increase the scheduled demand at the health care unit.” (Participant 4)

According to the Specialist Committee, the school setting proved capable of meeting the necessary requirements for implementation, and the experience was considered replicable throughout the network. Regarding the preparatory courses, a continuing education course entitled *Atraumatic Restorative Treatment: Concepts, Technique, Clinical Applications, and Work Process in Alternative Settings* was offered. The course was coordinated by the Departments of Orthodontics and Pediatric Dentistry, and Social Dentistry of the School of Dentistry of the University of São Paulo, and was offered to the entire oral health care network in São Paulo. A total of 2,400 oral health professionals (dentists, oral health technicians, and oral health assistants) participated.

The course was delivered in a hybrid format. Asynchronous content was made available on the Moodle platform, followed by a one-day in-person training course consisting of a theoretical phase (covering caries diagnosis concepts, ART indications, work processes, local planning, ergonomics, and biosafety) and a preclinical laboratory phase involving hands-on training in the ART technique.

For the in-person training, municipal management authorized professionals to travel to the School of Dentistry of the University of São Paulo without the need to compensate for time spent in training, provided that attendance was confirmed through attendance lists monitored by the health department team. Professionals who completed the course received certification from the university. According to the framework, these training activities addressed the domains of “needs” and “accessibility.”

With regard to strategy adoption, the Specialist Committee—particularly municipal management and regional support professionals—acted as champions for the strategy and played a central role in initiating the interventions. Their activities included sharing the pilot project experience, participating in planning, and joining the initial field actions. This group was also responsible for collecting and identifying the main questions and challenges faced by the Oral Health Teams and discussing them with the Specialist Committee to enable timely adjustments. Communication was coordinated through a WhatsApp group.

Regarding coordination with schools and preparation of the environment for clinical care, the Specialist Committee recommended prioritizing schools with closer ties to Basic Health Care Units, since preexisting relationships could facilitate implementation. These schools received a prior invitation for the dentists’ visit, along with identification of students classified as groups E or F according to the Demand Organization Index of the São Paulo State Department of Health—that is, students with at least one active cavitated carious lesion requiring restorative treatment.

The requirements for conducting treatment in classrooms included a well-lit space with a large, stable table on which children could lie during care. For children’s comfort, schools provided mats. The treatment room also had to be located as close as possible to a sink to allow handwashing between patients. In line with the framework, the school spaces were considered “adaptable” and “useful,” because they met all necessary criteria.

During the implementation stage, as shown in Table 5, a total of 10,300 children were treated and 23,638 teeth were restored. Unintentional (accidental) pulp exposure was reported in 314 teeth, corresponding to 1.3% of all procedures performed. The School Health Program ensured the feasibility of Artsus-SP, because it enabled the incorporation of ART into activities already conducted in school settings while fulfilling the requirements established during the training dissemination stage. After the first months of implementation, the strategy proved effective, expanding access to restorative treatment and oral health services.

**Table 5:** . Quantitative data after the first months of implementation of ART in the oral health care network of São Paulo, SP, Brazil.

Health technical supervision	No. of participating primary health care units	No. of students treated	No. of teeth treated	Mean number of teeth per treated student	Pulp exposure (n)
Central	5	137	443	2.5	2
East	93	3,074	6,996	2.3	84
North	47	1,162	2,764	2.6	64
West	20	526	1,048	2.0	11
Southeast	73	2,437	5,314	2.3	53
South	93	2,964	7,073	2.5	100
Total	331	10,3	23,638	2.3	314

However, several barriers and limitations were identified. Regional support professionals reported instances of resistance, including lack of resources (supplies and support materials) in some participating Basic Health Care Units; absence of School Health Program activities from contracts of Social Organizations (SOs), which are responsible for hiring and managing most of the city’s health workforce; lack of clinical protocols for managing cases with pulp exposure; and turnover among management personnel and oral health professionals.

## Discussion

The selection of ART was supported by the municipal health administration and by robust scientific evidence indicating that ART is a viable option for improving the oral health of schoolchildren in São Paulo. Randomized clinical trials have demonstrated that ART is not a temporary restoration and can be safely applied outside traditional dental clinics.^24,25^ However, only a few studies address the real-world application of ART within public health services.⁹ Implementation of such techniques in complex urban settings, such as São Paulo, requires contextual adaptation and strategic planning. This study helps clarify the steps that enabled the implementation of the Artsus-SP strategy, offering insights for replication or adaptation in other settings.

Unlike the conceptual model proposed by Fixsen et al.,^18^ the testing stage—typically associated with strategy adoption—was incorporated at the beginning of training dissemination. This decision reflected previous unsuccessful local attempts to implement ART, primarily due to its perception as a second-line or temporary treatment option.^26^ The pilot project highlighted both strengths and limitations of school-based care, confirming that schools could meet the requirements for safe, ergonomic, and biosafe procedures. The pre-existing School Health Program, implemented in 2007, facilitated integration. Nevertheless, resistance persisted due to negative prior experiences and perceptions of ART as less effective.^27-30^

Following the pilot project, the Specialist Committee reported increased optimism among oral health teams, driven by positive reception from schools and students (Table 4). This observation aligns with previous studies showing that children experience lower anxiety when treated at school compared with traditional clinical settings^20^. ART has also been associated with reduced anxiety when performed by generalists or oral health technicians.^20,31^ By the end of the pilot phase, the regional support professionals acted as the champions for the strategy, motivated by positive and practical experiences observed in the field. The preparatory courses incorporated evidence-based content alongside feedback from practice. Inclusion of hands-on training and official certification addressed the framework domains of “needs” and “accessibility” (Table 4). The hybrid model provided flexibility by combining asynchronous learning with targeted in-person sessions, supporting time management and effective training delivery.

**Table 4:** Training Dissemination: identified results and reference sources

Considered aspects (codes)	Identified results	Reference sources
Needs	Preparatory courses designed according to pilot project experiences; inclusion of challenges encountered during the work process in course content; provision of certification; practical activities	Meeting minutes; course syllabus (available on the Moodle platform)
Access	Flexibility in scheduling without disruption of regular work shifts through distance-learning (DL) phase; authorization for professionals to fulfill working hours at the School of Dentistry of the University of São Paulo on the day of the in-person activity	Meeting minutes

The WHO has acknowledged the persistent gap between academic knowledge and clinical practice^32^. The training courses were considered “accessible” and “compatible” with service routines, as defined by the framework, reinforcing the value of academic–public health partnerships in improving care^33^. With the pilot and training phases completed, the project entered the strategy adoption stage. According to the implementation framework^21^, professional adoption depends on leadership that supports and promotes the practice. In this context, the regional support professionals served as key leaders by disseminating the pilot experience, participating in planning, and reporting challenges to the university for rapid resolution.

This stage also demonstrated the potential for expanding access to care, particularly in underserved areas where students experience difficulties reaching health care units. Equity is a core principle of the Brazilian health system and essential to quality of life and dignity.^34,35^ Incorporating ART into programs targeting young children may also reduce the need for future invasive procedures^20,25^. During implementation, 10,300 children with 23,638 active cavitated carious lesions were treated within six months across 331 Basic Health Care Units. Many of these children likely had not adhered to prior referrals, a pattern frequently observed among more vulnerable populations, since absenteeism related to dental conditions (e.g., 22.5% of primary schoolchildren) has been reported as substantial in previous studies, reflecting persistent barriers to accessing care^13^. These children had previously been classified in groups E or F and were subsequently reclassified into group B. Analogous to the DMFT index, this shift corresponded to a reduction in the “decayed” component and an increase in the “filled” component. The overall DMFT remained unchanged; however, the redistribution of components (more restored teeth and fewer decayed teeth) indicated increased treatment coverage and improved access to care.

Regarding feasibility, schools proved to be strategic settings for care delivery, promoting collaboration between the health and education sectors and protective health determinants.^26,36^ Nevertheless, barriers such as high staff turnover and limited resources may hinder the continuity of implementation. Frequent turnover, combined with the fact that newly hired professionals often had not participated in the official training courses, emerged as a critical challenge. This gap can directly affect both the efficacy and efficiency of the strategy, since untrained professionals may be less prepared to perform ART procedures and to engage effectively in school-based activities. Addressing this issue through continuous or on-demand training programs would strengthen the long-term sustainability of the initiative. Overcoming these challenges remains essential for sustainable implementation.^37^

Quantitative data showed 314 cases (1.3%) of unintentional pulp exposure. In these cases, adherence to Ministry of Health clinical protocols was recommended, including protection of the pulp–dentin complex through direct pulp capping.^16^ In addition, with support from local coordinators, explanations were provided to teachers and parents or guardians, and care at the Basic Health Care Unit was prioritized in cases of pain or urgency. However, no formally published institutional protocol for facilitated access was available. Possible causes include diagnostic inaccuracies or technical limitations, because the literature emphasizes that diagnostic accuracy and operator experience are critical determinants of treatment success.^20,31,38,39^

To improve understanding of implementation dynamics, both barriers and clinical indicators were detailed, incorporating the perspectives of involved professionals. The staff reported challenges related to high turnover, variability in prior training, and occasional shortages of instruments and materials, which affected the consistency of care delivery. Clinical indicators provided quantitative evidence of both achievements and limitations. Professionals emphasized that hands-on practice, hybrid training, and feedback from field experience were essential for overcoming operational challenges. Based on these insights, practical recommendations for replicability include establishing continuous or on-demand training for newly hired staff, ensuring adequate resource allocation, maintaining close coordination between health care units and schools, and using clinical indicators to monitor quality and guide adaptive strategies in real-time.

An important limitation of this study was restricted access to primary data, since only consolidated datasets were available. This constraint precluded analytical statistical analyses and prevented precise identification of certain variables, such as the number of participating schools or individual-level caries outcomes. Although this limitation is substantial, it reflects a common challenge in implementation science, where routine service data are frequently aggregated and not fully accessible for research purposes. Whenever feasible, addressing this issue at the outset of implementation studies is recommended to enable more detailed analyses. Nonetheless, the comprehensive description of the implementation process, including barriers, facilitators, and contextual factors, provides valuable insights for evidence-informed decision-making. By documenting these processes, the study contributes to strengthening good practices in oral health policy and offers guidance for future strategies aimed at sustainable, context-adapted interventions.

In summary, motivated leadership and workforce engagement, local adaptations, a pilot phase, prior training, and academic–service integration were essential to the implementation of ART within São Paulo’s oral health network. The strategy improved access to care for children; however, overcoming structural barriers remains critical to ensure long-term sustainability.

## Conclusion

The implementation of ART within the ARTSUS-SP program yielded promising results. Key facilitators included motivated and well-qualified leadership and an engaged workforce, whereas high staff turnover and limited resources represented significant barriers, underscoring the need for continuous training and institutional support. Context-specific adaptations and collaboration between universities and municipal health services further contributed to the effectiveness and sustainability of the implementation.

## Data Availability

The datasets generated during and/or analyzed during the current study are available from the corresponding author on reasonable request.
